# Supplementary material for: Altered microRNA Transcriptome in Cultured Human Liver Cells upon Infection with Ebola Virus
Source: Int J Mol Sci. 2021 Apr 6;22(7):3792. doi: 10.3390/ijms22073792 (PMC8038836; doi:10.3390/ijms22073792)
Supplement: Supplementary file 1 [file ijms-22-03792-s001.zip › Supplementary_File/C_ GO_Analysis_Results/16-30nt_go_Mayinga-24h-Huh7_vs_Control-24h-Huh7_down.mature_mirna_targets/BP_FoldEnrichment.pdf]

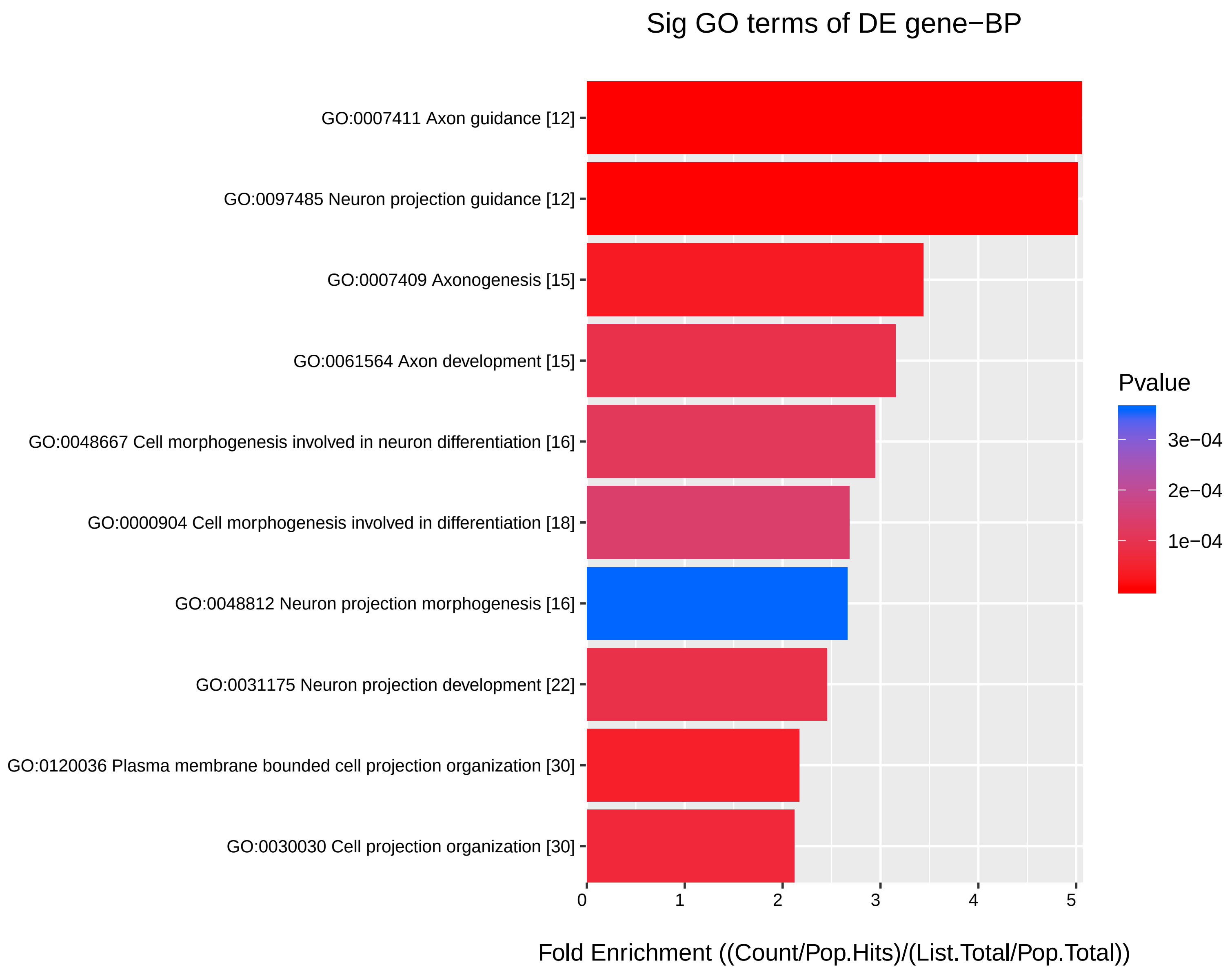
Sig GO terms of DE gene−BP
GO:0007411 Axon guidance [12]
GO:0097485 Neuron projection guidance [12]
GO:0007409 Axonogenesis [15]
GO:0061564 Axon development [15]
GO:0048667 Cell morphogenesis involved in neuron differentiation [16]
GO:0000904 Cell morphogenesis involved in differentiation [18]
GO:0048812 Neuron projection morphogenesis [16]
GO:0031175 Neuron projection development [22]
GO:0120036 Plasma membrane bounded cell projection organization [30]
GO:0030030 Cell projection organization [30]
0
1
2
3
4
5
Fold Enrichment ((Count/Pop.Hits)/(List.Total/Pop.Total))
Pvalue
3e−04
2e−04
1e−04
